# Time trends in case-mix and risk of revision following hip and knee arthroplasty in public and private hospitals: a cross-sectional analysis based on 476,312 procedures from the Dutch Arthroplasty Register

**DOI:** 10.2340/17453674.2024.40906

**Published:** 2024-06-17

**Authors:** Bart-Jan VAN DOOREN, Pelle BOS, Rinne M PETERS, Liza N VAN STEENBERGEN, Enrico DE VISSER, J Martijn BRINKMAN, B Willem SCHREURS, Wierd P ZIJLSTRA

**Affiliations:** 1 Department of Orthopedic Surgery, Medical Center Leeuwarden; 2 Department of Orthopedic Surgery, University Medical Center Groningen; 3 Department of Orthopedic Surgery, Martini Hospital, Groningen; 4 Dutch Arthroplasty Register (LROI), ‘s Hertogenbosch; 5 Department of Orthopedic Surgery, Canisius Wilhelmina Hospital, Nijmegen; 6 Department of Orthopedic Surgery, Kliniek Orthoparc Rozendaal; 7 Department of Orthopedic Surgery, Kliniek ViaSana, Mill; 8 Department of Orthopedic Surgery, Radboud University Medical Center, Nijmegen, the Netherlands

## Abstract

**Background and purpose:**

This study aims to assess time trends in case-mix and to evaluate the risk of revision and causes following primary THA, TKA, and UKA in private and public hospitals in the Netherlands.

**Methods:**

We retrospectively analyzed 476,312 primary arthroplasties (public: n = 413,560 and private n = 62,752) implanted between 2014 and 2023 using Dutch Arthroplasty Register data. We explored patient demographics, procedure details, trends over time, and revisions per hospital type. Adjusted revision risk was calculated for comparable subgroups (ASA I/II, age ≤ 75, BMI ≤ 30, osteoarthritis diagnosis, and moderate–high socioeconomic status (SES).

**Results:**

The volume of THAs and TKAs in private hospitals increased from 4% and 9% in 2014, to 18% and 21% in 2022. Patients in private hospitals were younger, had lower ASA classification, lower BMI, and higher SES compared with public hospital patients. In private hospitals, age and ASA II proportion increased over time. Multivariable Cox regression demonstrated a lower revision risk for primary THA (HR 0.7, CI 0.7–0.8), TKA (HR 0.8, CI 0.7–0.9), and UKA (HR 0.8, CI 0.7–0.9) in private hospitals. After initial arthroplasty in private hospitals, 49% of THA and 37% of TKA revisions were performed in public hospitals.

**Conclusion:**

Patients in private hospitals were younger, had lower ASA classification, lower BMI, and higher SES com­pared with public hospital patients. The number of arthroplasties increased in private hospitals, with a lower revision risk compared with public hospitals.

While public hospitals have traditionally been the primary providers of surgical procedures in the Netherlands, the significance of private hospitals in healthcare services has grown [1]. This is also notable in orthopedic procedures like total hip arthroplasty (THA), total knee arthroplasty (TKA), and unicompartmental knee arthroplasty (UKA), with an increasing number of these surgeries being conducted in private facilities in recent years [1]. This shift is driven by factors such as availability of services, extended waiting lists in public hospitals, and personal preferences of patients [2]. In addition to the growing need for arthroplasty, the COVID-19 pandemic had a strong impact on care, extending the waiting lists in public hospitals [2]. Efforts are being made to address the backlog of postponed surgeries in public hospitals; however, it may take some time towards full recovery. It is important to study the current situation of how and where the care for THA, TKA, and UKA patients is performed in the Netherlands.

This study aims to assess time trends in case-mix and to evaluate the risk of revision and causes following primary THA, TKA, and UKA in private and public hospitals.

## Methods

### Study design and data source

This study is a population-based cross-sectional study from the LROI. Since 2007, information on patient, procedure, and implant characteristics has been collected [1]. Currently, the LROI achieves a completion rate exceeding 97% for primary THA, TKA, and UKA [3]. The LROI bureau determines hospital types using public information. Though not formally validated, LROI’s annual report includes lists of university medical centers (UMCs), public hospitals, and private hospitals. Hospital types are confirmed annually by reviewing their classification and subsequently disclosed in the annual LROI report. This study is reported according to the STROBE guidelines [4].

### Data selection

**Figure 1:**
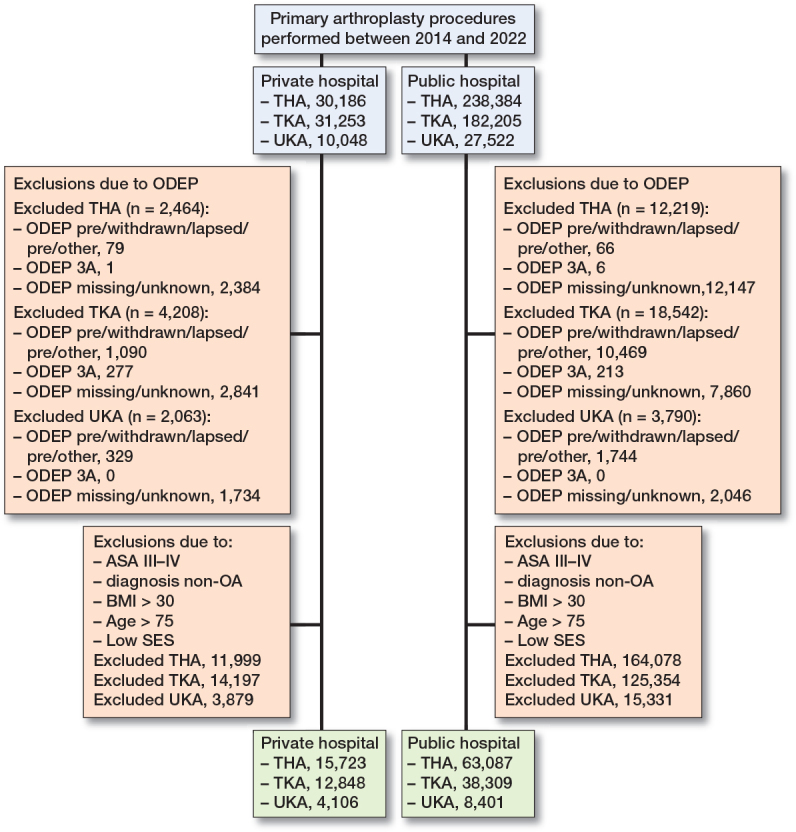
Flowchart of included primary THA procedures.

We included all primary THA, TKA, and/or UKA performed in private (n = 62,752) or public hospitals (n = 413,560) between January 1, 2014 and January 1, 2023. Prosthetic implants without a valid Orthopaedic Data Evaluation Panel (ODEP) rating, including those with a missing, expired, pre- or unknown ODEP category were excluded. Reliability of these ODEP implants cannot be assured, potentially impacting the validity of our results. Implants with an ODEP category < 5A were also excluded (Figure 1), as implants with a category < 5A are not permitted for general use in the Netherlands, except within the context of experimental studies. Primary procedures performed at university medical centers were not part of this dataset, as these patients form a non-representative group when compared with the general population.

### Variables

Patient (case-mix), procedure, and implant characteristics were obtained from the LROI. Case-mix is defined as factors describing population variation including age, gender, health condition (ASA, BMI), and socioeconomic status (SES). Data on SES was obtained from the Dutch Institute of Social Research, which calculated SES scores based on 4-number postal codes using average income, percentage of inhabitants with low income, percentage of unemployed residents, and education levels [5]. These scores were divided into quintiles at the 20th, 40th, 60th, and 80th percentiles. Subsequently, they were categorized into 3 groups: low SES (quintile 1), moderate SES (quintiles 2-4), and high SES (quintile 5). Information on vital status is received by linkage of data from the LROI and a national insurance database on health care (Vektis) [6]. Hospital type was defined as “private” or “public,” following the definition used by the LROI. Public hospitals are defined as healthcare facilities that are owned, operated, and funded by the government or a public entity. Private hospitals are defined as specialized healthcare facilities and are usually smaller independent providers that generally focus on 1 patient group, specialism, or treatment. The type of hospital performing revision was similarly categorized with the addition of “university medical center.”

### Outcomes

Primary outcomes included risk of revision for any reason, for infection, and the risk of minor or major revision in private and public hospitals. Revision arthroplasty is considered as a modification (exchange, addition, or removal) of 1 or more components of the original prosthesis [1]. Major revisions were characterized as revision of the acetabular and/or femoral component for THAs and revision of the femoral or tibial component of the TKA or UKA. Minor revisions were defined as inlay and/or femoral head exchange, inlay, and/or patella exchange or addition in TKA, and debridement antibiotics and implant retention (DAIR) procedures. Secondary outcomes included descriptive statistics covering patient and procedure characteristics, annual trends, and changes over time. In addition, type of hospital of revision, for patients who primarily received arthroplasty in a public or private hospital, was examined.

### Statistics

Patient and procedure characteristics and annual trends in private and public hospitals were expressed in numbers and percentages. Survival time was defined as time from primary arthroplasty to first revision arthroplasty for any reason, death or January 1, 2023 (end of follow-up). A crude cumulative incidence of revision was calculated for each hospital type using competing risk analyses, where death was considered to be a competing risk [7]. Multivariable Cox regression analyses were performed to calculate the risk of revision. To account for the significant differences in case-mix among hospital types, we specified a sub-selection of procedures. Procedures in patients with ASA I/II, age ≤ 75, BMI ≤ 30, OA diagnosis, and moderate to high SES were selected for survival analyses, as these patients are commonly treated in both types of hospitals. For all arthroplasties we adjusted for age, BMI, ASA class, and SES. Specifically for THA, we additionally adjusted for surgical approach. For TKA and UKA we added previous surgery as confounder into the model. Results were reported as hazard ratios (HR) with 95% confidence intervals (CI). The proportional hazard assumption for Cox models was assessed using scaled Schoenfeld residuals, which was not violated. For group comparisons, we used a chi-square test. For all tests, a 2-tailed significance level of P < 0.05 was used. SPSS statistics for Windows version 28.0 (IBM Corp ,Armonk, NY, USA) and R (R Foundation for Statistical Computing, Vienna, Austria) were used for statistical analyses.

### Ethics, data sharing, funding, and disclosures

The study was approved by the scientific advisory committee of the LROI. Ethical approval was not required according to the Dutch Medical Research involving Human Subjects Act (WMO) as all data is completely anonymous. Data was registered confidentially with patient consent. Sharing of data is not permitted by the LROI due to privacy regulations. No funding was received. The authors declared no conflicts of interest. Complete disclosure of interest forms according to ICMJE are available on the article page, doi: 10.2340/17453674.2024.40906

## Results

We included 253,887 primary THAs, 190,708 TKAs, and 31,717 UKAs (Figure 1). Prostheses with unknown, lapsed, pre-, missing, or ODEP category < 5A (THA 14,683; TKA 22,750; UKA 5,853) were excluded. In private hospitals, ODEP ratings were more frequently missing compared with public hospitals (THA 8% vs 5%; TKA, 9% vs 4 %; UKA 17% vs 7%). The median follow-up for THA, TKA, and UKA was 3.9, 4.1, and 3.1 years, respectively.

### Case-mix, procedure, and implant characteristics

Patients receiving THA, TKA, or UKA in private hospitals were generally younger, with lower BMI compared with patients from private hospitals (Tables 1–2). Also, patients from private hospitals generally had a lower ASA class and higher SES. The percentage of OA patients was 8% (CI 8.0–8.5) higher in private hospitals. In THA, the proportion of patients with larger femoral head sizes (36 mm) was 19% (CI 18.5–19.7) higher in private hospitals compared with public hospitals. The direct anterior approach (DAA) was preferred in private hospitals (59%), while the posterolateral approach (PLA) was favored in public hospitals (56%). In TKA, patellar components were 7% (CI 6.5–7.4) more commonly utilized in public hospitals than in private hospitals (Table 2).

**Table 1:** Patient and procedure characteristics of all primary total hip arthroplasties (THA) per type of hospital between 2014 and 2022. Values are count (%) and difference in percentage

Factor	Private hospital (n = 27,722)	Public hospital (n = 226,165)	Difference % (CI)
Age			
< 60	6,769 (24)	34,208 (15)	9.3 (8.7 to 9.8)
60–74	16,588 (60)	115,213 (51)	8.9 (8.3 to 9.5)
≥ 75	4,364 (16)	76,677 (34)	–18.2 (–18.6 to –17.7)
Female sex	17,460 (63)	147,730 (65)	–2.4 (–3.0 to –1.8)
ASA class			
I	9,354 (34)	32,294 (14)	19.5 (18.9 to 20.0)
II	17,884 (64)	143,050 (63)	1.3 (0.7 to 1.9)
III–IV	436 (1.6)	50,545 (23)	–20.8 (–21.0 to –20.6)
BMI			
< 18.5	140 (0.5)	2,067 (0.9)	–0.4 (–0.5 to –0.3)
18.5–25	11,201 (40)	73,830 (33)	7.8 (7.0 to 8.1)
25–30	11,757 (43)	92,344 (42)	1.2 (0.6 to 1.9)
30–35	4,105 (15)	40,175 (18)	–3.1 (–3.6 to –2.7)
35–40	275 (1.0)	11,188 (4.9)	–4.0 (–4.2 to –3.9)
> 40	23 (0.1)	2,803 (1.2)	–1.2 (–1.2 to –1.1)
Diagnosis OA	26,341 (95)	195,858 (87)	8.3 (8.0 to 8.5)
Previous operation	689 (2.5)	9,349 (4.1)	– 1.6 (–1.9 to –1.4)
Smoking	2,353 (8.5)	23,370 (11)	–2.0 (–2.4 to –1.7)
Charnley			
A	12,300 (45)	93,733 (44)	0.7 (0.1 to 1.3)
B1	8,556 (31)	63,292 (30)	1.4 (0.1 to 0.2)
B2	5,401 (20)	49,208 (23)	–3.5 (–4.0 to –3.0)
C	1,129 (4.1)	5,752 (2.7)	1.4 (1.2 to 1.7)
Socioeconomic status			
Low	3,156 (12)	47,140 (21)	–9.4 (–9.8 to –8.9)
Moderate	15,335 (57)	135,102 (60)	– 3.5 (–4.1 to –2.8)
High	8,430 (31)	41,330 (199	12.8 (12.3 to 13.4)
Fixation			
Cemented	1,074(3.9)	56,429 (25)	–21.2 (–21.5 to –20.9)
Cementless	25,662 (93)	144,178 (64)	28.6 (28.2 to 29.0)
Reverse hybrid	392 (1.4)	8,761(3.9)	–2.5 (–2.6 to –2.3)
Hybrid	544 (2.0)	15,584 (6.9)	–5.0 (–5.2 to –4.8)
Surgical approach			
Posterolateral	9,660 (35	125,939 (56)	–20.8 (–21.4 to –20.2)
Anterior	16,310 (59	65,373 (29)	29.9 (29.3 to 30.5)
Straight lateral	1,522 (5.5)	21,342 (9.4)	–3.9 (–4.2 to –3.7)
Direct superior	35 (0.1)	3,035 (1.3)	–1.2 (–1.3 to –1.2)
Other	195 (0.7)	10,476 (4.6)	–3.9 (–4.1 to –3.8)
Femoral head size			
22–28 mm	590 (2.2)	36,635 (16)	–14.2 (–14.4 to –14.0)
32 mm	15,824 (58)	139,586 (62)	–4.5 (–5.1 to –3.9)
36 mm	10,939 (40)	46,865 (21)	19.1 (18.5 to 19.7)
≥ 38 mm	0 (0.0)	818 (0.4)	–0.4 (–0.4 to –0.3)
Articulation ^b^			
CoC	3,141 (12)	11,070 (4.9)	6.6 (6.2 to 7.0)
CoM	2 (0.0)	155 (0.1)	–0.1 (–0.1 to –0.1)
CoP	17,669 (65)	142,748 (65)	0.6 (–0.1 to 1.2) ^a^
MoC	0 (0.0)	3 (0.0)	–0.0 (–0.0 to 0.0) ^a^
MoP	1,979 (7.3)	48,945 (22)	– 14.9 (–15.2 to –14.6)
ZoP	4,197 (16)	17,040 (7.7)	7.8 (7.4 to 8.3)
Acetabulum ODEP			
5A/5A*	1 (0.0)	1,951 (0.9)	–0.9 (–0.9 to –0.8)
7A/7A*	2,762 (10)	38,125 (17	–6.9 (–7.2 to –6.5)
10A/10A*	2,258 (8.1)	33,803 (15)	–6.8 (–7.2 to –6.4)
13A/13A*	6,028 (22)	31,283 (14)	7.9 (7.4 to 8.4)
15A/15A*	16,673 (60)	121,003 (53)	6.6 (6.0 to 7.2)
Femur ODEP			
5A/5A*	176 (0.6)	727(0.3)	0.3 (0.2 to 0.4)
7A/7A*	1,690 (6.1)	9,191 (3.6)	2.0 (1.7 to 2.3)
10A/10A*/10B	6,571 (24)	20,796 (8.7)	14.5 (14.0 to 15.0)
13A/13A*	1,531 (5.5)	22,361 (9.9)	–4.3 (–4.7 to –4.1)
15A/15A*	17,754 (64)	174,090 (77)	–12.9 (–13.5 to –12.3)

a Not significant.

b CoC = ceramic on ceramic; CoM = ceramic on metal; CoP = ceramic on polyethylene; MoC = metal on cobalt; MoP = metal on polyethylene; ZoP = zirconium on polyethylene.

BMI = body mass index.

OA = osteoarthritis

ODEP = Orthopaedic Data Evaluation Panel.

**Table 2:** Patient and procedure characteristics of all primary total knee (TKA) and unicompartmental knee arthroplasties (UKA) per type of hospital between 2014 and 2022. Values are count (%) and difference in percentage

Factor	TKA			UKA		
	Private hospital (n = 27,045)	Public hospital (n = 163,663)	Difference % (CI)	Private hospital (n = 23,732)	Public hospital (n = 7,985)	Difference % (CI)
Age						
< 60	5,910 (22)	23,131 (14)	7.7 (7.2 to 8.2)	2,597 (33)	6,710 (28)	4.2 (3.1 to 5.4)
60–74	17,169 (63)	90,050 (55)	8.5 (7.8 to 9.1)	4,632 (58)	13,547 (57)	0.9 (–0.1 to 2.1) a
≥ 75	3,963 (15)	50,431 (31)	–16.2 (–16.7 to –15.7)	756 (9.5)	3,472 (15	–5.2 (–5.9 to –4.4)
Female sex	15,519 (57)	105,323 (64)	–7.0 (–7.6 to –6.4)	4,187 (53)	13,114 (55)	–2.8 (–4.1 to –1.6)
ASA class						
I	7,278 (27)	15,866(9.7)	17.3 (16.7 to 17.8)	2,607 (33)	3,559 (16)	17.7 (16.6 to 18.8)
II	18,963 (70)	108,217 (66)	4.0 (4.3 to 4.6)	5,078 (64)	16,053 (68)	–3.9 (–5.1 to –2.7)
III–IV	760 (2.8)	39,390 (24)	–21.3 (–21.6 to –21.0)	283 (3.6)	4,111 (17)	–13.8 (–14.4 to –13.2)
Diagnosis OA	26,260 (97)	158,167 (97)	0.4 (0.2 to 0.6)	7,872 (99)	23,355 (99	0.2 (–0.1 to 0.4) ^a^
Previous operation	9,723 (37)	41,944 (26)	11.0 (10.4 to 11.6)	2,171 (28)	5,346 (23)	4.6 (3.5 to 5.7)
Smoking	2,054 (7.7)	12,857 (8.2)	–0.4 (–0.8 to –0.1)	653 (8.3)	2,189 (9.5)	–1.2 (–1.9 to –0.4)
BMI						
< 18.5	39 (0.1)	262 (0.2)	–0.1 (–0.1 to 0.1) ^a^	5 (0.1)	14 (0.1)	–0.01 (–0.001 to 0.001) ^a^
18.5–25	6,047 (22)	26,681 (17)	6.0 (5.4 to 6.5)	1,764 (22)	4,072 (18)	4.9 (3.9 to 6.0)
25–30	12,795 (48)	64,679 (40)	7.5 (6.9 to 8.2)	3,746 (47)	10,092 (43)	4.3 (3.1 to 5.6)
30–35	7,034 (26)	44,841 (28)	–1.6 (–2.2 to –1.1)	2,048 (26)	6,544 (28)	–2.0 (–3.1 to –0.8)
35–40	761 (2.8)	18,325 (11)	–8.5 (–8.8 to –8.3)	278 (3.5)	2,166 (9.3)	–5.7 (–6.4 to –5.2)
> 40	152 (0.6)	6,245 (3.9)	–3.3 (–3.4 to –3.2)	49 (0.6)	502 (2.1)	– 1.5 (–1.8 to –1.3)
Charnley						
A	11,367 (43)	63,591 (39)	3.2 (2.6 to 3.9)	3,757 (48)	12,134 (52)	–3.7 (–5.0 to –2.4)
B1	9,167 (34)	55,658 (35)	–0.1 (–0.7 to 0.5) ^a^	2,616 (34)	6,977 (30)	3.7 (2.5 to 4.9)
B2	5,106 (19)	36,290 (23)	–3.3 (–3.9 to –2.8)	1,279 (16)	4,031 (17)	–0.8 (–1.7 to –0.1)
C	937 (3.5)	5,335 (3.3)	0.2 (–0.0 to 0.2) ^a^	162 (2.1)	294 (1.3)	0.8 (0.4 to 1.2)
Socioeconomic status						
Low	3,582 (14)	34,678 (22)	–7.7 (–8.3 to –7.4)	1,027 (13)	4,353 (19)	–5.3 (–6.2 to –4.4)
Moderate	15,331 (58)	97,108 (60)	–1.6 (–2.2 to –0.9)	4,475 (58)	14,600 (62)	–4.4 (–5.6 to –3.1)
High	7,285 (28)	29,760 (18)	9.4 (8.8 to 10.0)	2,224 (29)	4,488 (19)	9.6 (8.5 to 10.8)
Fixation						
Cemented	24,276 (90)	153,908 (94)	–4.3 (–4.7 to –3.9)	2,355 (30)	10,876 (46)	–16.3 (–17.5 to –15.2)
Cementless	1,635 (6.0)	5,344 (3.3)	2.8 (2.5 to 3.1)	5,628 (70)	12,843 (54)	16.4 (15.2 to 17.6)
Hybrid	1,134 (4.2)	4,411 (2.7)	1.5 (1.2 to 1.7)	2 (0.0)	13 (0.1)	–0.01 (–0.01 to 0.01) ^a^
Surgical approach						
Medial parapatellar	26,265 (97)	156,447 (96)	1.6 (1.4 to 1.8)	7,692 (97)	21,953 (93)	4.0 (3.5 to 4.5)
Lateral parapatellar	89 (0.3)	1,244 (0.8)	–0.4 (–0.5 to –0.4)	104 (1.3)	145 (0.6)	0.7 (0.4 to 1.0)
Vastus	584 (2.2)	4,822 (3.0)	–0.8 (–1.0 to –0.6)	150 (1.9)	1,464 (6.2)	–4.3 (–4.7 to –3.9)
Other	30 (0.1)	849 (0.5)	–0.4 (–0.5 to –0.4)	5 (0.1)	108 (0.4)	–0.4 (–0.5 to –0.3)
Patella component						
Yes	3,840 (14)	34,604 (21)	–6.9 (–7.4 to –6.5)	n.a.	n.a.	n.a.
No	23,205 (86)	129,059 (79)	6.9 (6.5 to 7.4)	7,985 (100)	23,732 (100)	n.a.
Type of femur						
Posterior stabilized	16,225 (60)	97,739 (60)	0.3 (–0.4 to 0.9) ^a^	n.a.	n.a.	n.a.
Minimally stabilized	10,774 (40)	62,808 (38)	1.5 (0.8 to 2.1)			
Other	46 (0.2)	3,116 (1.9)	–1.7 (–1.8 to –1.7)			
Tibia mobility						
Fixed	25,223 (93)	143,781 (88)	5.3 (5.0 to 5.6)	2,349 (29)	10,821 (46)	–16.2 (–17.4 to –15.0)
Mobile	n.a.	n.a.	n.a.	5,627 (71)	12,834 (54)	16.4 (15.2 to 17.6)
Rotating	1,822 (6.7)	19,657 (12)	–5.3 (–5.6 to –5.0)	9 (0.1)	77 (0.3)	–0.2 (–0.3 to –0.1)
ODEP						
5A/5A*/5B	902 (3.3)	2,319 (1.4)	1.9 (1.7 to 2.1)	11 (0.1)	515 (2.2)	–2.0 (–2.2 to –1.8)
7A/7A*	5,803 (22)	14,970 (9.1)	12.3 (11.8 to 12.8)	0 (0.0)	0 (0.0)	0
10A/10A*/10B	6,566 (24)	45,570 (28)	–3.6 (–4.1 to –3.0)	0 (0.0)	204 (0.8)	–0.9 (–1.0 to –0.7)
13A/13A*	3,636 (13)	45,390 (28)	–14.3 (–14.8 to –13.8)	7,592 (95)	15,177 (64)	31.1 (30.4 to 31.9)
15A/15A*	10,138 (38)	55,414 (34)	3.6 (3.0 to 4.2)	382 (4.8)	7,836 (33)	–28.2 (–29.0 to –27.5)

a Not significant.

BMI = body mass index.

OA = osteoarthritis

ODEP = Orthopaedic Data Evaluation Panel.

### Trends in case-mix over time

**Figure 2:**
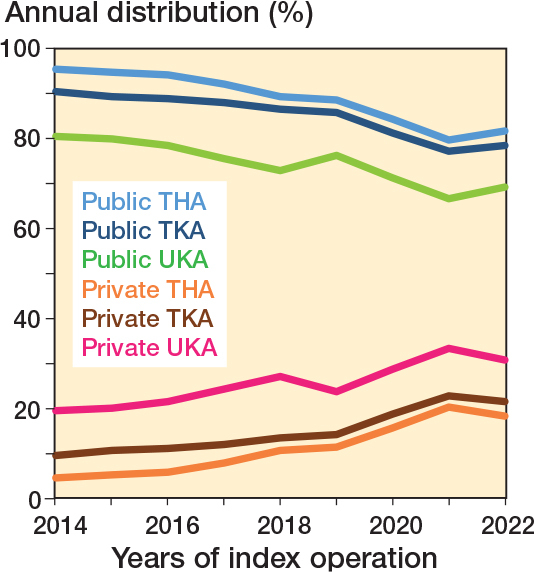
Distribution (%) of primary THA, TKA, and UKA in the Netherlands between 2014 and 2022 according to hospital type (university medical centers not included).

Between 2014 and 2022, the proportion of primary procedures performed in private hospitals gradually increased from 4% to 18% for THAs, 9% to 21% for TKAs, and 19% to 31% for UKAs (Figure 2).

In recent years (2020–2022), private hospitals showed an increase in the proportion of patients aged > 75 years for THA (+5%, CI 3.9–5.6), TKA (+4%, CI 3.3–5.1), and UKA (+4%, CI 2.3–4.9). In contrast, public hospitals observed minimal change. Private hospitals demonstrated a shift towards a higher proportion of ASA-II patients (THA: +8%, CI 7.0–9.2; TKA: +9%, CI 6.7–7.8; UKA: +13%, CI 10.5–14.8), while public hospitals observed an increase in patients with ASA III/IV (THA: +9%, CI 8.9–9.7; TKA: +10%, CI 9.7–10.7; UKA: +7%, CI 6.2–8.2) (Tables 3 and 4, see Appendix).

**Table 3:** Patient and procedure characteristics of all primary THAs per type of hospital according to period. Values are count (%)

	Private hospital		Public hospital	
Factor	2014–2019	2020–2022	2014–2019	2020–2022
Age				
< 60	3,119 (26)	3,650 (23)	23,532 (15)	10,676 (15)
60–74	7,487 (61)	9,101 (59)	78,833 (52)	36,380 (51)
≥ 75	1,599 (13)	2,765 (18)	50,253 (33)	26,424 (34)
Female sex	7,613 (62)	9,847 (63)	100,123 (66)	73,466 (65)
ASA class				
I	4,682 (38)	4,672 (30)	24,111 (16)	8,183 (11
II	7,333 (6)0	10,551 (68)	98,810 (65)	44,240 (60)
III–IV	187 (1.5)	249 (1.6)	29,497 (19)	21,048 (29)
Diagnosis OA	11,454 (94	14,887 (96)	133,197 (87)	62,661 (86)
BMI				
< 18.5	67 (0.6)	73 (0.5)	1,351 (0.9)	716 (1.0)
18.5–25	4,886 (40)	6,315 (41)	49,144 (33)	24,686 (34)
25–30	5,279 (44)	6,478 (42)	62,865 (42)	29,479 (41)
30–40	1,825 (17)	2,555 (16)	34,679 (23)	16,684 (23)
> 40	17 (0.1)	6 (0.0)	1,855 (1.2)	948 (1.3)
Fixation				
Cemented	661 (5.4)	413 (2.7)	39,095 (26)	17,334 (24)
Cementless	10,935 (90)	14,727 (95)	97,391 (64)	46,787 (64)
Reversed hybrid	249 (2.0)	143 (0.9)	6,510 (4.3)	2,251 (3.1)
Hybrid	343 (2.8)	201 (1.3)	8,820 (5.8)	6,764 (9.2)
Surgical approach				
Posterolateral	5,044 (41)	4,616 (30)	88,627 (58)	37,312 (5)1
Anterior	5,885 (48)	10,425 (67)	36,708 (24)	28,655 (39)
Straight lateral	1,135 (9.3)	387 (2.5)	18,680 (12)	2,662 (3.6)
Direct superior	2 (0.0)	33 (0.2)	785 (0.5)	2,250 (3.1)
Other	140 (1.1)	55 (0.4)	7,884 (5.2)	2,592 (3.5)
Femoral head size				
22–28 mm	245 (2.0)	345 (2.2)	28,806 (19)	8,029 (11)
32 mm	6,983 (58)	8,841 (58)	91,961 (61)	47,625 (65)
36 mm	4,789 (40)	6,150 (40)	30,052 (20	16,813 (23)
≥ 38 mm	0 (0.0)	0 (0.0)	490 (0.3)	328 (0.5)
Articulation ^a^				
CoC	1,208 (10)	1,933 (13)	8,877 (6.0)	2,193 (3.1)
CoM	0 (0.0)	2 (0.0)	17 (0.0)	138 (0.2)
CoP	7,649 (65)	10,020 (66)	93,104 (62)	49,644 (70)
MoC	0 (0.0)	0 (0.0)	3 (0.0)	0 (0.0)
MoP	593 (5.0)	1,386 (9.2)	36,827 (25)	12,118 (17)
ZoP	2,409 (20)	1,788 (12)	10,205 (6.8)	6,835 (9.6)

a For abbreviations, see Table 1.

**Table 4:** Patient and procedure characteristics of all primary TKA and UKA per type of hospital according to period. . Values are count (%)

Factor	TKA				UKA			
	Private hospital		Public hospital		Private hospital		Public hospital	
	2014–2019	2020–2022	2014–2019	2020–2022	2014–2019	2020–2022	2014–2019	2020–2022
Age								
< 60	3,520 (24)	2,390 (19)	16,878 (15)	6,253 (13	1,343 (38)	1,254 (28)	4,066 (30)	2,644 (26)
60–74	9,271 (63)	7,898 (64)	63,623 (55)	26,427 (54	1,900 (54)	2,732 (61)	7,759 (57)	5,788 (57)
≥ 75	1,866 (13)	2,097 (17)	34,228 (30)	16,203 (33	261 (7.4)	495 (11)	1,758 (13)	1,714 (17)
Female sex	8,338 (57)	7,181 (58)	74,338 (65)	30,985 (63	1,873 (53)	2,314 (52)	7,616 (56)	5,498 (54)
ASA class								
I	4,411 (30)	2,867 (23)	12,416 (11)	3,450 (7.1)	1,353 (39)	1,254 (28)	2,372 (18)	1,187 (12)
II	9,769 (67)	9,194 (75)	78,056 (68)	30,161 (62)	1,983 (56)	3,095 (69)	9,270 (68)	6,783 (77)
III–IV	472 (3.2)	288 (2.3)	24,141 (21)	15,269 (31)	165 (4.7)	118 (2.6)	1,937 (14)	2,174 (11)
Diagnosis OA	14,236 (97)	12,024 (98)	110,952 (97)	47,215 (97)	3,451 (99)	4,421 (99)	13,373 (99)	9,982 (99)
BMI								
< 18.5	24 (0.2)	15 (0.1)	187 (0.2)	75 (0.2)	2 (0.1)	3 (0.1)	10 (0.1)	4 (0.0)
18.5–25	3,236 (22)	2,811 (23)	18,289 (16)	8,392 (17)	717 (21)	1,047 (24)	2,283 (17)	1,789 (18)
25–30	6,933 (48)	5,862 (48)	45,312 (40)	19,367 (40)	1,638 (48)	2,108 (47)	5,733 (43)	4,359 (43)
30–40	4,194 (29)	3,601 (29)	44,315 (40)	18,851 (39)	1,031 (30)	1,295 (29)	5,017 (38)	3,693 (37)
> 40	131 (0.9)	21 (0.2)	4,447 (4.0)	1,798 (3.7)	43 (1.3)	6 (0.1)	281 (2.2)	211 (2.1)
Fixation								
Cemented	13,943 (95	10,333 (84	106,984 (93	46,924 (96	1,119 (34	1,156 (26	7,150 (53	3,726 (37
Cementless	113 (0.8)	1,522 (12	4,563 (4.0)	781 (1.6)	2,304 (66	3,324 (74	4,625 (47	6,418 (63
Hybrid	604 (4.1)	530 (4.3)	3,231 (2.8)	1,18 (2.4)	1 (0.0)	1 (0.0)	11 (0.1)	2 (0.1)
Surgical approach								
Medial parapatellar	14,041 (96)	12,224 (99)	109,859 (96)	46,588 (95)	3,317 (95)	4,375 (98)	12,618 (93)	9,335 (92)
Lateral parapatellar	64 (0.4)	25 (0.2)	985 (0.9)	259 (0.5)	59 (1.7)	45 (1.0)	105 (0.8)	40 (0.4)
Vastus	526 (3.6)	58 (0.5)	3,639 (3.2)	1,183 (2.4)	114 (3.3)	46 (0.8)	812 (6.0)	652 (6.4)
Other	4 (0.0)	26 (0.2)	92 (0.1)	757 (1.6)	1 (0.0)	4 (0.1)	19 (0.1)	89 (0.9)
Tibia mobility								
Fixed	13,746 (94)	11,477 (93)	99,557 (87)	44,224 (90)	1,191 (34)	1,158 (26)	7,091 (52)	3,730 (37)
Mobile	n.a.	n.a.	n.a.	n.a.	2,304 (66)	3,323 (74)	6,495 (48)	6,410 (63)
Rotating	914 (6.2)	908 (7.3)	15,025 (13)	4,632 (10)	n.a.	n.a.	n.a.	n.a.
Unicondylar side								
Medial	n.a.	n.a.	n.a.	n.a.	3,256 (92)	4,351 (99)	12,594 (99)	9,505 (99)
Lateral	n.a.	n.a.	n.a.	n.a.	54 (1.6)	51 (1.2)	89 (0.7)	47 (0.5)
Patellar component	2,088 (14)	1,752 (14)	25,048 (2)2	9,556 (20)	n.a.	n.a.	n.a.	n.a.
Type of femur								
Posterior stabilized	8,000 (55)	8,225 (66)	66,279 (58)	31,460 (64)	n.a.	n.a.	n.a.	n.a.
Minimally stabilized	6,616 (45)	4,158 (34)	46,589 (40)	16,219 (33)	n.a.	n.a.	n.a.	n.a.
Other	44 (0.3)	2 (0.0)	1,910 (1.7)	1,206 (2.5)	n.a.	n.a.	n.a.	n.a.

### Risk of revision for all causes

**Figure 3:**
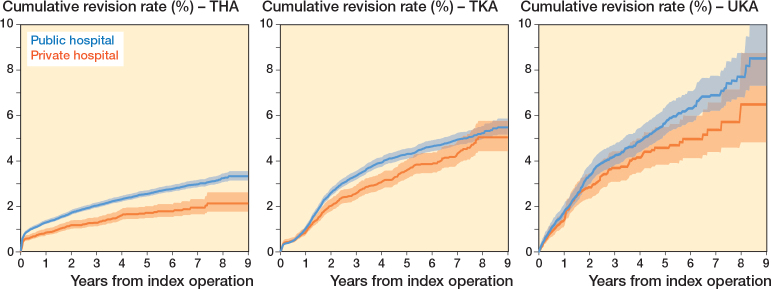
Crude cumulative incidence of revision for any reason in primary TKA, TKA, and UKA per type of hospital for patients with ASA I/II, BMI ≤ 30, age ≤ 75, moderate or high SES. and OA.

After subgroup selection, we observed lower 1-, 3-, 5-, and 7-year crude cumulative revision rates of THA in private hospitals compared with public hospitals (Figure 3; Table 5, see Appendix). For TKA, 3- and 5-year revision rates were also lower in private hospitals (Figure 3; Table 5, see Appendix). Crude cumulative revision rates for UKA were comparable between the 2 hospital types (Figure 3; Table 5, see Appendix). Multivariable Cox regression analysis demonstrated a lower risk of revision for primary THA (HR 0.8, CI 0.7–0.9), TKA (HR 0.8, CI 0.7–0.9), and UKA (HR 0.8, CI 0.6–1.0) in private hospitals compared with public hospitals (Table 6). An overview of reasons for revision showed that, for THA, infection rates were lower in private hospitals compared to public hospitals (0.4% vs. 0.6%, respectively; p = 0.02). Similarly, dislocations, loosening of femur, and loosening of acetabulum were less frequent in private hospitals compared with public hospitals (Table 7). For TKA, there were no differences in infections, periprosthetic fractures, loosening of tibia, and loosening of femur between the 2 types of hospitals (Table 8). For UKA, private hospitals had comparable rates of infection, periprosthetic fracture, instability, loosening of tibia, loosening of femur, and inlay wear (Table 8).

**Table 5:** Crude cumulative incidence of revision in primary TKA, TKA, and UKA per type of hospital for patients with ASA I/II, BMI < 30, age < 75, moderate or high SES, and osteoarthritis

Follow-up	Crude cumulative incidence (%) of revision			
	Private hospital		Public hospital	
	At risk	% (CI)	At risk	% (CI)
THA				
1-year	13,989	0.7 (0.6–0.8)	58,896	1.0 (1.0–1.1) ^a^
3-year	7,834	1.2 (1.0–1.4)	45,874	1.9 (1.8–2.0) ^a^
5-year	4,215	1.7 (1.4–1.9)	31,861	2.4 (2.3–2.6) ^a^
7-year	1,683	1.9 (1.6–2.2)	16,851	2.9 (2.7–3.0) ^a^
TKA				
1-year	11,667	0.5 (0.4–0.6)	35,994	0.5 (0.4–0.5)
3-year	7,445	2.4 (2.1–2.7)	27,958	3.0 (2.8–3.2) ^a^
5-year	4,686	3.3 (2.9–3.7)	19,648	4.1 (3.9–4.4) ^a^
7-year	2,519	4.1 (3.7–4.6)	10,385	4.8 (4.5–5.1)
UKA				
1-year	3,585	0.8 (0.6–1.2)	7,590	1.0 (0.8–1.3)
3-year	1,905	3.4 (2.8–4.1)	5,173	3.9 (3.5–4.4)
5-year	1,041	4.5 (3.7–5.4)	3,130	5.3 (4.7–5.9)
7-year	472	5.2 (4.2–6.3)	1,460	6.8 (6.1–7.6)

a Statistically significant difference between private and public hospitals

**Table 6:** Multivariable survival analysis of revision for any reason in primary THA, TKA, and UKA per type of hospital for patients with ASA I/II, BMI < 30, age < 75, moderate or high SES, and diagnosis osteoarthritis

Hospital	Total number	Revisions	Hazard ratio (CI)	
			Crude	Adjusted
THA ^a^				
Private	15,723	205	0.7 (0.6–0.8) ^d^	0.8 (0.7–0.9) ^d^
Public	63,087	1,513	1.0	1.0
TKA ^b^				
Private	12,848	348	0.8 (0.8–0.9) ^d^	0.8 (0.7–0.9) ^d^
Public	38,309	1,453	1.0	1.0
UKA ^c^				
Private	4,106	129	0.8 (0.7–1.0)	0.8 (0.6–0.98) ^d^
Public	8,401	379	1.0	1.0

a THA: Adjusted for age, BMI, ASA class, SES, surgical approach.

b TKA: Adjusted for age, BMI, ASA class, SES, previous surgery.

c UKA: Adjusted for age, BMI, ASA class, SES, previous surgery.

d P < 0.05.

**Table 7:** Reasons for revision for THA, subdivided between private and public hospitals between 2014 and 2022 for patients with ASA I/II, BMI ≤ 30, age ≤ 75, moderate or high SES, and OA. Values are count (%). A patient may have more than 1 reason for revision

Reason	Private hospital n = 15,723	Public hospital n = 63,087	P value
Infection	67 (0.4)	365 (0.6)	0.02
Periprosthetic fracture	42 (0.3)	191 (0.3)	0.5
Dislocation	38 (0.2)	383 (0.6)	< 0.001
Loosening of femur	28 (0.2)	294 (0.5)	< 0.001
Loosening of acetabulum	19 (0.1)	137 (0.2)	0.02
Cup/liner wear	3 (0.0)	30 (0.0)	0.1
Other	39 (0.2)	305 (0.5)	< 0.001

**Table 8:** Reasons for revision for TKA and UKA, subdivided between private and public hospitals between 2014 and 2022 for patients with ASA I/II, BMI ≤ 30, age ≤ 75, moderate or high SES, and OA. Values are count (%). A patient may have more than 1 reason for revision

Reason	TKA Private hospital n = 12,772	UKA Public hospital n = 38,309	P value	Private hospital n = 7,985	Public hospital n = 23,732	P value
Infection	76 (0.6)	262 (0.7)	0.3	16 (0.4)	34 (0.4)	0.9
Periprosthetic fracture	0 (0.0)	20 (0.1)	0.01	7 (0.2)	13 (0.2)	0.8
Instability	121 (0.9)	444 (1.2)	0.04	29 (0.7)	87 (1.0)	0.1
Loosening of tibia	68 (0.5)	238 (0.6)	0.2	25 (0.6)	52 (0.6)	0.8
Loosening of femur	24 (0.2)	87 (0.2)	0.4	6 (0.1)	14 (0.2)	0.9
Inlay wear	12 (0.1)	26 (0.1)	0.4	9 (0.2)	15 (0.2)	0.6
Malalignment	42 (0.3)	193 (0.5)	0.01	17 (0.4)	44 (0.5)	0.4
Arthrofibrosis	27 (0.2)	142 (0.4)	0.01	1 (0)	7 (0.1)	0.2
Patellar pain	88 (0.7)	456 (1.2)	< 0.001	12 (0.3)	30 (0.4)	0.6
Patellar dislocation	7 (0.1)	39 (0.1)	0.1	0 (0)	2 (0)	0.3
Other	49 (0.4)	154 (0.4)	0.8	44 (0.6)	195 (0.8)	0.1

### Risk of revision for infection, minor and major revisions

Multivariable Cox regression analysis demonstrated no difference in the risk of revision for infection for primary THA (HR 0.9, CI 0.7–1.2), TKA (HR 0.9, CI 0.7–1.2), and UKA (HR 0.9, CI 0.5–1.7) in private hospitals compared with public hospitals. The adjusted risk of minor revision was lower in private hospitals for all arthroplasties compared with public hospitals (Table 9). The adjusted risk of major revision was lower in private hospitals for THA (HR 0.8, CI 0.7–0.9), but not for TKA (HR 0.9, CI 0.7–1.0) and UKA (HR 0.9, CI 0.7–1.2).

**Table 9:** Multivariable survival analysis of revision for infection, minor or major revisions in primary THA, TKA, and UKA per type of hospital for patients with ASA I/II, BMI < 30, age < 75, moderate or high SES, and diagnosis osteoarthritis

Hospital	Total number	Revisions	Hazard ratio (CI)	
			Crude	Adjusted
THA ^a^				
For infection				
Private	15,723	67	0.8 (0.6–1.1)	0.9 (0.7–1.2)
Public	63,087	365	1.0	1.0
Minor				
Private	15,723	54	0.6 (0.4–0.8) ^d^	0.7 (0.5–0.95) ^d^
Public	63,087	393	1.0	1.0
Major				
Private	15,723	147	0.7 (0.6–0.8) ^d^	0.8 (0.7–0.9) ^d^
Public	63,087	1,106	1.0	1.0
TKA ^b^				
For infection				
Private	12,848	76	1.0 (0.7–1.2)	0.9 (0.7–1.2)
Public	38,309	262	1.0	1.0
Minor				
Private	12,843	185	0.8 (0.7–0.9) ^d^	0.8 (0.7–0.9) ^d^
Public	38,283	807	1.0	1.0
Major				
Private	12,843	159	0.9 (0.8–1.1)	0.9 (0.7–1.0)
Public	38,283	629	1.0	1.0
UKA ^c^				
For infection				
Private	4,106	16	1.1 (0.4–1.9)	0.9 (0.5–1.7)
Public	8,401	34	1.0	1.0
Minor				
Private	4,106	48	0.7 (0.5–1.0)	0.7 (0.5–0.9) ^d^
Public	8,401	155	1.0	1.0
Major				
Private	4,106	80	0.9 (0.7–1.2)	0.9 (0.7–1.2)
Public	8,401	221	1.0	1.0

a THA: Adjusted for age, BMI, ASA class, SES, surgical approach.

b TKA: Adjusted for age, BMI, ASA class, SES, previous surgery.

c UKA: Adjusted for age, BMI, ASA class, SES, previous surgery.

d P < 0.05.

### Type of hospital for revision

The majority (95–96%) of patients initially treated in public hospitals received their revision arthroplasty in a public hospital. In contrast, when primary arthroplasty was performed in a private hospital, 48% of THA revisions, 37% of TKA revisions, and 22% of UKA revisions were performed in another type of hospital (Table 10). This shift was mainly seen for major revisions (Table 11, see Appendix).

**Table 10:** Type of hospital of revision according to type of hospital of primary THA, TKA, and UKA procedure. Values are count (%)

Type of hospital of revision procedure	Type of hospital of primary procedure	
	Private hospital	Public hospital
THA		
Private hospital	220 (52)	25 (0.4)
Public hospital	171 (40)	6,384 (96)
University medical center	35 (8.2)	246 (3.7)
TKA		
Private hospital	466 (63)	152 (2.5)
Public hospital	237 (32)	5,655 (95)
University medical center	37 (5.0)	174 (2.9)
UKA		
Private hospital	207 (78)	40 (3.5)
Public hospital	56 (21)	1,094 (95)
University medical center	4 (1.5)	16 (1.4)

**Table 11:** Type of revision procedures for THA, TKA, and UKA in context of hospital switch for revision procedure. Values are count (%)

	Primary procedure at private hospital			Primary procedure at public hospital		
	Revised at public	Revised at private	Revised at UMC	Revised at private	Revised at public	Revised at UMC
THA						
Revisions	171	220	35	25	6,384	246
Minor revision ^a^	25 (15)	81 (37)	6 (17)	7 (28)	2,001 (31)	8 (3.3)
Major revision ^b^	144 (84)	126 (57)	29 (83)	16 (64)	4,185 (66)	227 (92)
Missing	2 (1.2)	13 (5.9)	0 (0.0)	2 (8.0)	198 (3.1)	11 (4.5)
TKA						
Revisions	237	466	37	152	5,655	174
Minor revision ^a^	76 (32)	295 (63)	10 (27)	67 (44)	3,136 (55)	32 (18)
Major revision ^b^	143 (60)	144 (31)	26 (70)	76 (50)	2,300 (41)	136 (78)
Missing	18 (7.6)	27 (5.8)	1 (2.7)	9 (5.9)	219 (3.9)	6 (3.4)
UKA						
Revisions	56	207	4	40	1,094	16
Minor revision ^a^	18 (32)	84 (41)	1 (25)	7 (83)	374 (34)	0 (0.0)
Major revision ^b^	33 (59)	116 (56)	3 (75)	33 (17)	675 (62)	15 (94)
Missing	5 (8.9)	7 (3.4)	0 (0.0)	0 (0.0)	45 (4.1)	1 (6.3)

a Minor revision is defined as revision of the inlay and/or femoral head, or inlay and/or patellar exchange or addition.

b Major revision is defined as revision of the acetabular and/or femoral component for THAs and revision of the femoral or tibial component of the TKA or UKA.

UMC = University Medical Center.

## Discussion

The aim of our study was to assess time trends in case-mix and to evaluate the risk of revision and causes following primary THA, TKA, and UKA in private and public hospitals in the Netherlands. We hypothesized that patients treated in private hospitals are relatively healthier with fewer comorbidities and lower BMI compared with patients treated in public hospitals. Consequently, we expect that, when similar subgroups treated in both hospitals are compared, the revision rates would be comparable between both types of hospitals We found a significant rise in the proportion of patients treated in private hospitals in the Netherlands in recent years. Furthermore, significant differences in case-mix were observed between patients from private hospitals compared with patients from public hospitals. For patients who had the primary arthroplasty in a private hospital, a significant number of revisions were performed in public hospitals or university medical centers. After subgroup selection, we found a lower risk of revision for all examined arthroplasties in patients from private hospitals compared with patients from public hospitals. These results must be interpreted cautiously, as unregistered confounding factors in registry data may affect outcomes.

Historically, concerns have been raised regarding outsourcing healthcare services to private providers potentially compromising the standard of care [8]. For instance, a study discovered that the heightened outsourcing of NHS services to private providers from 2013 to 2020 was associated with lower quality of patient care and elevated rates of deaths from treatable causes [8]. Also, previous research regarding orthopedic procedures from earlier periods revealed elevated revision rates for hip and knee replacements performed in private hospitals, casting doubt on their capacity to deliver high-quality healthcare [9-11]. As the private sector has become more professionalized, there has been a concerted effort to address earlier concerns through stringent regulatory frameworks. This is shown in more recent studies from various countries, which reported on the performance of private and public hospitals. A study from the Australian national arthroplasty registry reported higher revision rates for THA and TKA in patients treated in private hospitals [12]. However, the authors found no difference after controlling for implant choice, suggesting that the difference in revision rates was explained by the choice of implant, rather than the type of hospital. A study from England found that private providers, who tended to provide hip or knee replacements to healthier patients, had better outcomes when compared with public providers, even after adjustment for preoperative differences [13]. In addition, a retrospective study using the Norwegian Patient Register reported that private non-profit hospitals had significantly lower readmission rates compared with public hospitals among patients receiving THA [14]. While these studies are in principle comparable to our study, the healthcare systems may vary substantially between countries, which poses challenges in the comparison of results.

We found a clear difference in case mix for patients treated in private compared with public hospitals. Private hospitals predominantly treated younger patients with lower BMI and ASA class, primarily for OA. Previous international studies evaluated “cherry-picking” behavior among private hospitals [15-16]. It is suggested that private hospitals tend to treat less complex patients than public hospitals [15-16]. A contributing factor to this patient selection in private hospitals in the Netherlands is the guidance provided by the Dutch health care inspectorate [17]. According to these guidelines, private hospitals are advised to refrain from treating patients classified as ASA III/IV. This restriction leads to a certain selection of patients directed to private hospitals. This was confirmed in our data, as 97–98% of THA, TKA, or UKA patients in private hospitals had ASA I/II, and 96–99% of patients had a BMI < 35. Therefore, our data indicates that private hospitals adhere well to health inspection regulations, demonstrating a selective approach in treating patients.

The influence of case-mix on the rate of revision for hip and knee arthroplasties is well established in the literature. Hence, patient selection should be considered when interpreting revision rates of private and public hospitals. For instance, the a priori revision risk is significantly increased in patients with higher BMI and ASA class [18-22]. Notwithstanding our efforts to narrow the selection criteria to patients with ASA I/II, age ≤ 75, BMI ≤ 30, a diagnosis of OA, and moderate to high SES, in order to create equal groups, we observed a lower risk of revision among patients from private hospitals. We believe that, despite this subgroup selection, the disparities in patients between private and public hospitals may not be entirely addressed, and unobserved residual confounding may impact the results. For example, patients in public hospitals often present with comorbidities associated with a higher risk of revision (e.g., uncontrolled diabetes), which is not fully captured in registry data. Moreover, while ASA class is useful for preoperative assessment, it may not fully describe a patient profile. LROI registry data contain the diagnosis but lack information concerning the complexity of a surgical procedure. Patients with complex hip and knee conditions may face a higher risk of revision due to the inherent anatomical challenges. It is possible that private hospitals handle fewer surgeries of a complex nature, based on surgeon selection. For example, more complex conditions like hip dysplasia, abnormal anatomical morphology, post-traumatic injuries, bone deformities, and joint instability might be more frequently addressed in public hospital settings. However, we were unable to verify this with our data. Moreover, it could be possible that surgeons in private hospitals, where revision surgeries are less frequent compared with their counterparts in public hospitals, may possess a higher threshold for performing revision procedures. This could be attributed to factors such as lower caseloads, disparities in resource availability, and variances in access to multidisciplinary support teams. However, this is an assumption that we are unable to validate with our data and it is important to note that while this observation may apply to some smaller private clinics, it may not represent the situation in larger private hospitals, where revisions are part of the normal workflow. In addition, it is worth noting that in the Netherlands many surgeons work in both private and public settings, mitigating the perception of a threshold.

The lower revision rates observed in private hospitals can partially be explained by the difference in procedure-related factors. The most prominent difference in the risk of revision between private and public hospitals was observed in primary THAs. We observed a higher percentage of large femoral heads and utilization of the DAA in private hospitals. Previous studies reported lower dislocation rates when larger femoral heads are used [23]. Moreover, lower dislocation rates have been reported for the DAA compared with the PLA [23-26]. In addition, the LROI 2022 annual report demonstrated a lower 13-year risk of revision for any reason for the DAA (3.9%, CI 3.4–4.5) compared with the PLA (5.6%, CI 5.4–5.7) [1].

Increased surgical volume and surgeon experience have been associated with lower revision rates and may play a role in the difference in revision rates between private and public hospitals [27-29]. Private hospitals, often specializing in certain treatments, tend to have higher surgical volume in their area of expertise. As a result, surgeries are typically performed by experienced surgeons, potentially resulting in lower revision rates. Moreover, the absence of orthopedic residents in private hospitals may contribute, as some studies report higher revision rates for residents [30]. However, other research suggests that residents performing total joint arthroplasties under the guidance of experienced consultant colleagues achieve outcomes comparable to those of senior surgeons [31-33].

### Limitations

First, due to the observational study design, this study lacks the ability to control for all confounding variables. Second, our data does not contain information regarding early postoperative complications that did not necessitate revision arthroplasty. Third, we stated that increased surgical volume and surgeon experience are associated with lower revision rates. However, we were unable to examine this with our data. Lastly, revision rates do not fully reflect the overall quality of care. Other quality indicators like patient satisfaction, readmission rates, mortality rates, rehabilitation progress, quality of postoperative care, and costs were not considered.

### Conclusion

In the Netherlands, a significant rise is seen in arthroplasty procedures performed in private hospitals. Different case-mix is seen in private and public hospitals, with private hospitals predominantly treating younger, lower BMI, and relatively healthier patients. A lower risk of revision for all examined arthroplasties was seen in private hospitals.

### In perspective

It is likely that private hospitals will continue to fill a substantial part of the capacity gap in the upcoming years. The shift towards private hospital care is safe in terms of revision rates, on the premise of proper patient selection and backup facilities. Hence, the observed trend is well justified. It is suspected that the more comorbid and hence higher risk patients may be left to wait for their surgery in public hospitals. In addition, possible failed primary arthroplasty from a private hospital may need to be revised elsewhere, again increasing the waiting lists. Therefore, higher revision rates in public hospitals, in combination with the increased case mix complexity, emphasize the necessity for increased resource allocation and funding. Hence, directing more healthcare resources and funding to public hospitals may be necessary, particularly for complex patient needs.

## Notes

Authors contributed to: (1) study design and study protocol, (2) gathered data, (3) analyzed data, (4) initial draft, and (5) final draft. BvD and PB: 1–5; RMP and WPZ: 1, 2, 4, 5; LNvS, EdV, JMB, and BWS: 1, 2, 5.

The authors thank the Dutch Orthopaedic Association (NOV) and the LROI for granting access to this database and for offering valuable perspectives on this manuscript.

Handling co-editors: Marianne Westberg and Robin Christensen

*Acta* thanks Wender Figved and Lene Bergendal Solberg for help with peer review of this manuscript.
